# The Terrell Medical Society

**Published:** 1890-03

**Authors:** 


### THE TERRELL MEDICAL SOCIETY.

Dr. F. S. White, President, in the chair. Dr. Fowler reported a case of uterine hemorrhage which notwithstanding the administration of astringents, failed to procure its arrest. Dr. Callagan was called in and not obtaining the consent of the patient to make an examination, agreed with Dr. Fowler to prescribe bi. chl. sol. ferri as a wash and Donavan's sol. internally; the hemorrhage was arrested and patient's condition greatly improved.

Dr. Jones thought it a manifestation of scrofula, and that the hemorrhage was the result of ulceration and that the above treatment effected a cure.

Dr. Smiley held that continued hemorrhage from the uterus was a symptom of grave lesion and should incite a thorough examination as it will commonly demonstrate the presence of polypi, or some such character of excresenses, which should be removed by surgical means. Dr. Monday agreed with Dr. Smiley as to the importance of making a thorough examination; generally in all such cases you will find either polypus or vegetative growths which must be removed to effect a cure. Dr. Monday held with Dr. Smiley the great importance in making a thorough examination in any and all uterine troubles, and treating them skillfully, yet boldly. Dr. Garrett reported the condition of a patient suffering from what he supposed to be a tumor in the hepatic region of the colon.

Dr. Stroud asked if he was certain there was a tumor, and if he was, he was certainly very derelict in duty in neglecting to aspirate at once, as it is through such negligence that many succumb.

Dr. Nelson reported a case of stenosis of the cervix; patient had been married several years, with no offspring. Having made a thorough examination, and found this condition existing, at once resorted to mechanical dilatation of the cervical canal, and so successful was the operation that in a short time, patient became pregnant and is now a happy mother.

Dr. Strain remarked that several such cases had fallen into his hands and was convinced that the favorable dilatation of the cervical canal, in such cases as the one just reported by Dr. Nelson was the most rational treatment, and one followed by the greatest benefit in dysmenorrhœa in all its varied forms.

Dr. Nelson asked if primiparæ did not usually suffer more or less laceration.

Dr. Tidwell thought they generally do.

Dr. Inabnit said he had examined numerous cases and had failed to discover any laceration whatever existing.

Dr. Strain said he could speak from experience, and was not prepared to say that the condition of the atmosphere or that of his surroundings tended to favor it but it was generally the case in his extended practice.

